# Supplementary material for: Starvation resistance in the nematode Pristionchus pacificus requires a conserved supplementary nuclear receptor
Source: Zoological Lett. 2024 Mar 13;10:7. doi: 10.1186/s40851-024-00227-y (PMC10938818; doi:10.1186/s40851-024-00227-y)
Supplement: Supplementary file 1 — Supplementary Material 1 [file 40851_2024_227_MOESM1_ESM.docx]

**Supplementary Information**

**Sup. Tab. 1. Overrepresentation analysis shows no bias towards any given chromosomal location among targets of *Ppa-*NHR-10.** The 37 candidate regulatory targets (DEGs) of *Ppa-*NHR-10, which were identified in the condition-specific RNA-seq experiments, were used as input for the analysis. No significant bias (FDR-corrected *P*-value < 0.05) towards any particular chromosome was found for *Ppa-*NHR-10’s target genes. DEG = differentially expressed gene, FDR = false discovery rate.

| **chromosome** | **number_of_genes** | **number_of_DEGs** | **fold_enrichment** | **FDR_corrected_pvalue** |
| --- | --- | --- | --- | --- |
| Chr1 | 6869 | 5 | 0.5684764688986556 | 0.35319808452624085 |
| Chr2 | 4462 | 11 | 1.9253031606236448 | 0.13589186241137188 |
| Chr3 | 3736 | 5 | 1.045199374963829 | 0.9702253905888654 |
| Chr4 | 6594 | 10 | 1.1843690824582545 | 0.8351614949834785 |
| Chr5 | 4097 | 5 | 0.9531034573748757 | 1 |
| ChrX | 3002 | 1 | 0.26015089039739275 | 0.35319808452624085 |

**Sup. Tab. 2. Loss of *Ppa-nhr-10* does not affect the expression levels of *P. pacificus*’s orthologs of the *C. elegans* propionate shunt genes.** El Paco ver. 3 annotations were used for *P. pacificus*. *s*-values indicate FSOS event probability; significant changes to the expression levels (*s* < 0.005) could not be observed in any case. Strain indicated the *Ppa-nhr-10* mutant that was compared to wild-type worms. Cel = *C. elegans*, FSOS = false sign or smaller than 50% up- or down-regulated, l2fc = log_2_-fold change in expression levels, mmse = minimum mean squared error, sd = standard deviation.

| **Cel_gene** | **pristionchus_ortholog** | **l2fc_mmse** | **l2fc_posterior_sd** | **s-value** | **strain** | **treatment** |
| --- | --- | --- | --- | --- | --- | --- |
| alh-8 | PPA18546 | 0.3120081894467676 | 0.183586125982154 | 0.779593708146979 | RS3920 | fed |
| alh-8 | PPA18546 | 0.0700641209645215 | 0.124433208963777 | 0.903335595238679 | RS3920 | starved |
| alh-8 | PPA18546 | 0.1578326679985086 | 0.116470311709115 | 0.870830237769447 | RS3921 | fed |
| alh-8 | PPA18546 | 0.1310214980190643 | 0.111534703830993 | 0.801497484867020 | RS3921 | starved |
| ech-6 | ppa_stranded_DN19950_c1_g2_i1 | 0.2915677450017385 | 0.121252839902834 | 0.890590474805354 | RS3920 | fed |
| ech-6 | ppa_stranded_DN19950_c1_g2_i1 | 0.0278618218738554 | 0.058205088196759 | 0.922220261931968 | RS3920 | starved |
| ech-6 | ppa_stranded_DN19950_c1_g2_i1 | 0.302048528212008 | 0.099893413969880 | 0.865990199881571 | RS3921 | fed |
| ech-6 | ppa_stranded_DN19950_c1_g2_i1 | 0.1002545176406774 | 0.078147156473995 | 0.831089635382915 | RS3921 | starved |
| hach-1 | ppa_stranded_DN24485_c0_g1_i1 | 0.1503250384307045 | 0.179955991152806 | 0.871633632978635 | RS3920 | fed |
| hach-1 | ppa_stranded_DN24485_c0_g1_i1 | -0.003755268928726 | 0.048962247587748 | 0.916236852972432 | RS3920 | starved |
| hach-1 | ppa_stranded_DN24485_c0_g1_i1 | 0.3403493778772363 | 0.171220920053186 | 0.685735755852485 | RS3921 | fed |
| hach-1 | ppa_stranded_DN24485_c0_g1_i1 | -0.029993946051769 | 0.095674037390754 | 0.816727072214799 | RS3921 | starved |
| hphd-1 | PPA05876 | 0.2710472432776699 | 0.186482563130857 | 0.845357698346291 | RS3920 | fed |
| hphd-1 | PPA05876 | -0.021954386415356 | 0.066963132113470 | 0.912373249230267 | RS3920 | starved |
| hphd-1 | PPA05876 | 0.1715677303340060 | 0.120611075832775 | 0.868252620804282 | RS3921 | fed |
| hphd-1 | PPA05876 | -0.388037641153159 | 0.170339234415574 | 0.558929874614430 | RS3921 | starved |
